# Exclusive enteral nutrition: An optimal care pathway for use in adult patients with active Crohn's disease

**DOI:** 10.1002/jgh3.12256

**Published:** 2019-09-10

**Authors:** Alice Day, Jessica Wood, Sarah Melton, Robert V Bryant

**Affiliations:** ^1^ Department of Gastroenterology, The Queen Elizabeth Hospital Adelaide South Australia Australia; ^2^ Department Nutrition and Dietetics, The Alfred Melbourne Victoria Australia

**Keywords:** Crohn's disease, diet, exclusive enteral nutrition, nutrition

## Abstract

**Background and Aim:**

Exclusive enteral nutrition (EEN) is progressively being used as a therapeutic option for adults with Crohn's Disease (CD); however, there is no standardized approach to delivering this therapy. The aim of this study is to develop an optimal care pathway for using EEN in adults with CD. This will create a standard of care that can be used as a benchmark practice and will provide direction for future research.

**Methods:**

A working group of 12 multidisciplinary inflammatory bowel disease specialists across Australia and New Zealand was convened to develop a practical, clinically focused care pathway for using EEN in adults with active CD. Six key areas were identified as part of the care pathway: clinical indications, nutrition assessment, EEN protocol, monitoring, accessing formula, and food reintroduction. Current literature was identified via systematic review, and quality of evidence was graded. Consensus expert opinion was used where literature gaps were identified.

**Results:**

An optimal care pathway for using EEN in adults with CD was developed with six key consensus statements on how to use EEN in adults with active CD. These key statements identify clinical indications for use, nutrition assessment, enteral prescription and duration of therapy, monitoring criteria, food reintroduction, and the role of partial EEN. An accompanying patient resource was also developed.

**Conclusion:**

EEN is recommended as a treatment option to induce remission in adults with active CD. The consensus statements developed are practical and are based on best available evidence and expert opinion to assist in developing a standardized approach to delivering EEN therapy.

## Graphical abstract

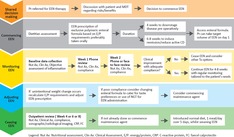

Exclusive enteral nutrition (EEN) is an alternative therapeutic option to corticosteroids for patients with Crohn's disease (CD). This study aimed to develop an optimal care pathway for using EEN in adults with active CD. Six key consensus statements regarding the practical application of EEN in adults with active CD were developed based on available evidence and expert opinion to assist clinicians in delivering this therapy.

## Introduction

The role of exclusive enteral nutrition (EEN) in the management of adult patients with active Crohn's disease (CD) is evolving. The basic premise of efficacy is the same as pediatric patients, yet few studies have been conducted on the adult cohort. This difference has been attributed to the lack of access to a multidisciplinary team, perceived intolerance and noncompliance in adults, and a lack of experience amongst inflammatory bowel disease (IBD) physicians.^1^ Subsequently, clinical guidelines for using EEN therapy in adults with active CD have not been developed.

Although high‐quality studies are limited, and existing study findings are heterogeneous, EEN may offer similar efficacy to corticosteroids for induction of remission in adults with CD.^2^, ^3^ EEN has advantages over corticosteroids not only via avoidance of steroid‐related side effects but through improvement in nutritional indices, endoscopic mucosal healing, and treatment of IBD‐related complications such as strictures and fistulae, leading to surgical avoidance.^1^, ^4^, ^5^, ^6^

In pediatric IBD populations, the evidence base for EEN is stronger. EEN is globally considered a first‐line treatment for remission induction in children with CD (60–80%).^7^ In adults, in contrast to other western countries, only Japan recommends EEN as a first‐line treatment, with 80% remission rates reported.^7^, ^8^, ^9^, ^10^, ^11^, ^12^, ^13^ Use of EEN in adults therefore warrants further exploration as the mechanism of EEN in both adult and pediatric CD populations is likely to be the same.

In tertiary IBD centers in Australia and New Zealand, a standardized approach to delivering EEN therapy is lacking. International IBD standards of care guidelines provide little guidance.^8^, ^9^ Therefore, the aim of this study was to develop an optimal care pathway for using EEN in adults with active CD to facilitate use of and standardize EEN therapy in this cohort.

## Methods

A working group of nine IBD‐specialist dietitians and three IBD‐specialist gastroenterologists from Australia and New Zealand was created through an expression of interest to the Australasian Society of Parenteral and Enteral Nutrition (AuSPEN) and Dietitians Crohn's and Colitis Australian Network members. Clinicians were defined as adult IBD experts if they were currently working in IBD services in Australia and New Zealand where EEN therapy is used.

### *Development of key summary statements and an optimal care pathway*

Six key areas for an optimal care pathway were identified by group consensus: clinical indications, nutrition assessment, EEN protocol, monitoring, accessing formula, and food reintroduction. The expert working group was then split into six groups. Each group conducted literature searches of Medline, Embase, and Cochrane Library databases between June and October 2018 using the following MeSH terms: inflammatory bowel diseases, Crohn's disease, enteral nutrition, nutrition therapy, nutritional support, diet therapy, diet, nutrition assessment, and malnutrition.

Key summary statements were developed for each area of the care pathway by reviewing literature quality using National Health and Medical Research Council, Oxford Centre for Evidence‐based Medicine Levels of Evidence, and Melynyk and Fineout‐Overholt hierarchies of evidence.^14^ Gaps were identified where absence of literature or consensus opinion made it difficult in a clinical setting to translate evidence into practice.

A patient resource detailing instructions for following an EEN protocol was developed and reviewed by the expert working group and a Victorian patient advisory group.

## Results

**Figure 1:**
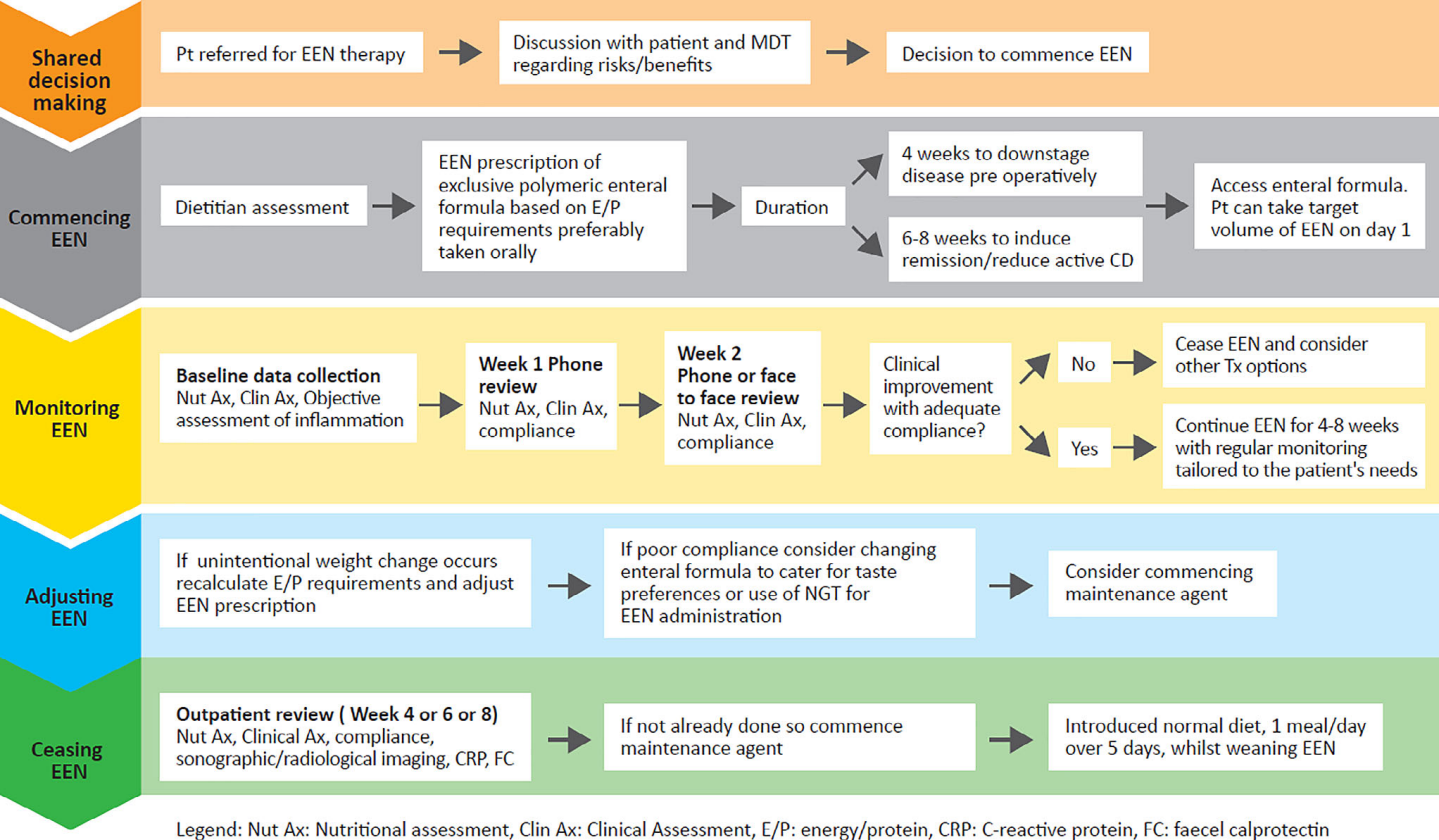
Optimal care pathway for using exclusive enteral nutrition in adults with active CD. CD, Crohn's disease; Clin Ax, clinical assessment; CRP, C‐reactive protein; E/P, energy/protein; EEN, exclusive enteral nutrition; FC, fecal calprotectin; MDT, multidisciplinary team; NGT, nasogastric tube; Nut Ax, nutritional assessment.

The optimal care pathway (Fig. 1) guides a step‐by‐step approach to using EEN therapy in adults with active CD. The key summary statements provide practical guidance for EEN therapy. The accompanying patient resource can be used by clinicians for patient education and is available on the AuSPEN website (http://www.auspen.org.au).

### *Statement 1: Clinical indications for EEN therapy in adults with active CD*

1. EEN is likely to be as effective as corticosteroids for remission induction in adults with CD who are able to tolerate EEN therapy (level 3 evidence).
2. EEN may be effective in managing complicated CD, including enterocutaneous fistulae and inflammatory strictures (level 4 evidence).
3. EEN may downstage CD severity prior to surgery and improve surgical outcomes or obviate the need for surgery (level 3 evidence).

#### *Remission induction*

Trials reporting on EEN for the induction of remission in adult patients with CD report variable results and are of low quality, with limitations discussed elsewhere.^2^, ^3^ Data demonstrating that EEN is more effective than corticosteroids in inducing remission are stronger in pediatric populations. Clinical remission has been induced in 60–86% of patients, accompanied by improvement in biomarkers of inflammation C‐reactive protein (CRP) and fecal calprotectin (FC).^2^, ^15^ Pediatric data also show that EEN achieves mucosal healing and has comparable efficacy to antitumor necrosis alpha therapy in children with CD; however, there are no comparative studies in adults.^16^, ^17^

A narrative review of seven studies evaluating EEN compared to corticosteroids in adults with CD found that EEN is likely to be as effective as corticosteroids when tolerated (23–100% remission rate *vs* 30–100% in corticosteroid group).^3^ In contrast, a Cochrane review found that EEN was inferior to corticosteroids, even by per‐protocol analysis (risk ratio 0.82, 95% confidence interval [CI] 0.70–0.95).^2^ The inability of adults to tolerate EEN's palatability was identified as the primary reason for high rates of nonadherence and withdrawal from trials.^3^ Recent studies show promise, with a prospective trial showing that even 2 weeks of EEN in adults with active CD was sufficient to yield significant improvements in symptoms, CRP, and FC.^18^ There is evidence that EEN may be more effective in those with newly diagnosed CD and those with ileal disease location; however, further studies are required to substantiate these observations.^3^

#### *Optimization/avoidance of surgery*

Around 70% of patients with CD develop stricturing or penetrating complications requiring surgery within 20 years of diagnosis.^19^ Presurgical optimization is important given that mortality rates are higher in patients with IBD who undergo emergency, rather than elective, surgery.^20^ Malnutrition and a high inflammatory burden have also been associated with higher rates of surgical complications in CD.^21^, ^22^

There is emerging evidence for the role of preoperative EEN in improving surgical outcomes for patients with complicated CD. In a retrospective case‐controlled study, 51 adult patients with stricturing or penetrating CD received preoperative EEN^4^; 94% tolerated more than 4 weeks of preoperative EEN (mean 6.3 weeks) and had a shorter length of surgery with significantly fewer surgical complications than control patients who went straight to surgery (8 *vs* 32%, *P* < 0.001). A significant decrease in CRP was also observed. There was a ninefold increase in the incidence of postoperative abscess and/or anastomotic leak (odds ratio 9.1; 95% CI 1.2–71.2, *P* = 0.04) in the control group. This study also showed that EEN may facilitate avoidance of surgery all together. Of patients who received EEN, 25% successfully progressed to immunomodulator therapy without surgical intervention.^4^ Similar rates of surgical avoidance have been described in older studies of patients with CD.^23^, ^24^

In another retrospective study of 114 patients, an average of 3 weeks of presurgical EEN reduced anastomotic leakage and the need for a diverting stoma.^25^ A randomized controlled trial of 91 patients reported that preoperative nutritional optimization with EEN reduced inflammatory markers within 4 weeks (mean 25 days).^5^ No significances in surgical outcomes were reported in this study as the capacity to do so was limited by power.

#### *Managing complications*

Small studies have shown the efficacy of EEN for management of CD complications. In a group of adult patients with inflammatory stricturing CD (*n* = 59), EEN administered for 12 weeks achieved radiological remission of stricturing disease in 54% and resolution of clinical symptoms in 65% of patients.^26^ Similarly, in patients with enterocutaneous fistulae, two small studies have shown that 12 weeks of EEN led to fistula closure in 62.5–75% of patients.^27^, ^28^ In those with intra‐abdominal collections, use of EEN for over 12 weeks was associated with resolution of collection in 76% of patients.^28^ There are no data on EEN for perianal CD in adults, although there are several case reports describing efficacy in pediatric patients.^29^

#### *Nutritional support*

EEN may be used to improve nutritional status in malnourished adult patients with CD, which is an important consideration prior to surgery.^5^ In contrast to metrics of inflammation that may improve within 2 weeks, a noticeable change in albumin and body weight is likely to require at least 6–8 weeks of EEN.^4^, ^5^

### *Statement 2: Workup prior to EEN therapy in adults with active CD*

1. The goals of EEN therapy should be tailored to the clinical indication for use of EEN and should be established prior to commencing therapy (level 7 evidence).
2. Patients commencing EEN should have a comprehensive nutritional assessment to inform nutrition prescription and monitoring (level 7 evidence).

#### *When to use EEN therapy*

EEN should be available as a primary therapy for adults with CD.^8^ The clinical indications for using EEN therapy should inform shared decision‐making and setting goals for EEN prescription. New diagnosis, clinical response to medications, disease location, disease complications, patient experience, and need for surgical intervention should also be considered.^3^

#### *Nutrition screening and assessment*

Malnutrition among IBD patients is well documented and linked to adverse outcomes.^21^, ^30^, ^31^ Patients with active CD or those who have had multiple bowel resections are at a higher risk of poor nutritional status and nutrient deficiencies.^32^, ^33^ Causation can be complex and multifactorial, including inadequate dietary intake (poor appetite, food avoidance, prolonged fasting), malabsorption, gastrointestinal losses, disease activity including catabolic effect of inflammation, steroid‐induced catabolism, increased nutritional requirements, surgical resections, or drug–food interactions.^31^

As part of establishing goals for EEN therapy, patients should be screened for malnutrition risk and should undergo a comprehensive nutritional assessment by a dietitian.^1^, ^34^ A detailed examination of metabolic, nutritional, and functional variables (including anthropometric, biochemical, clinical, dietary, and social history) should inform an initial nutrition prescription. This management plan should be monitored to prevent or treat nutritional deficiencies.^30^

### *Statement 3: Prescribing EEN therapy in adults with active CD*

1. A polymeric whole‐protein formula is recommended for EEN (level 2 evidence).
2. EEN should be available to all IBD patients via health‐care subsidy programs or pharmacies (level 7 evidence).
3. No other table food or fluids are recommended for consumption when following an EEN regimen except for water (level 4 evidence).
4. The duration of EEN regimen should be determined by the preidentified clinical goals (level 7 evidence).
5. EEN can be commenced at target volume on day 1 (level 7 evidence).
6. EEN should be ceased if there is failure to respond within 2 weeks (level 7 evidence).
7. EEN regimens may need individualization to promote adherence (level 7 evidence).

#### *Choosing a formula*

Formula choice should be informed by established efficacy and tolerability (Table 1). Multiple meta‐analyses showed no difference in efficacy between EEN formulas, whether they be polymeric, elemental, or have an altered fat content.^2^, ^3^, ^7^ The benefits of a polymeric formula include palatability, cost, variety, availability, and the ability to be administered both orally and enterally. Brand‐specific [(Modulen (Nestle Health Science, Epalinges, Switzerland), Osmolite (Abbott Australasia, Macquarie Park NSW, Australia), Paediasure (Abbott Australasia, Macquarie Park NSW, Australia), and Ensure Plus (Abbott Australasia, Macquarie Park NSW, Australia)] and generic EEN formulas have been trialed. Juice‐based polymeric formulas have not been reported.^2^, ^3^, ^18^, ^35^ This may be because juice‐based formulas tend to be higher in osmolarity, lower in protein, and lack fat‐soluble vitamins.

**Table 1:** Promoting adherence to exclusive enteral nutrition therapy: Practical considerations

Consideration	Rationale
Patient preference	Formula choices should be individualized to taste, palatability, and volume; should provide variety; and should meet nutrition prescription
Availability and access	Establish whether formula can be accessed via hospital subsidized program or purchased from local pharmacy
Affordability	Offer the most cost‐efficient way of supplement provision to promote compliance
Route	Oral polymeric formulas are most versatile as they can be administered orally and enterally if nasogastric feeding is indicated
Convenience	Offer ready‐made formulas and powdered options in different volumes to promote compliance
Nutritional content	Formula choices should meet the nutrition prescription with consideration of osmotic load and absorption

#### *Consuming formula*

EEN should be encouraged orally, with the option of nasogastric administration if patients are unable to tolerate adequate oral volumes.^2^, ^3^, ^13^

#### *Access to formula*

EEN formulas are available through health‐care subsidy programs or privately (online retailer or pharmacy). Providing patients with formula options and samples can assist in promoting compliance. Out‐of‐pocket expenses are likely to vary between individual services depending on whether site‐specific supplement programs are available or health‐care subsidies exist.

#### *Consumption of other fluids or foods*

There is inconsistent practice in the consumption of other fluids and food items with EEN. Survey data suggest it is common practice to include other nonnutritive food and drink items, such as chewing gum, broth, tea, or coffee, with EEN.^36^ However, most current studies evaluating the efficacy of EEN in CD have not detailed any recommendations for nonprescribed fluid or food intake. Of three studies providing such detail (two pediatric and one adult), one allowed polymeric formula and water only (80.5% complete remission), one allowed additional nonnutritive gums and sweets (85% remission rate), and the adult study allowed tea (remission rate not reported).^18^, ^28^, ^35^, ^37^

#### *Duration of therapy*

Variable timeframes of EEN therapy have been evaluated in clinical trials of adult patients with CD, ranging from 10 days to beyond 12 weeks. Accordingly, guidelines for the duration of EEN therapy for remission induction in adults are inconsistent.^3^ Any clear direction for appropriate duration of EEN is further confused by the goals of therapy, whether they are remission induction, objective amelioration of inflammation, presurgical optimization, or resolution of complicated disease.

The optimal duration of EEN therapy therefore remains unclear. Current data suggest that duration should be tailored to clinical indication (Table 2). The likelihood of patient adherence should be taken into account to avoid waning with protracted EEN prescription.^3^

**Table 2:** Clinical indication and duration of EEN therapy

Clinical indication for EEN therapy	Recommended duration of EEN therapy†
Induction of remission	6–8 weeks
Bridge to medical therapy	4–12 weeks until maintenance therapy is within therapeutic range
Preoperative EEN	Minimum of 4 weeks
Management of abdominal abscess or fistula	6–12 weeks with monitoring to direct duration of therapy

† Recommendation derived from systematic review of existing literature combined with expert consensus opinion.

EEN, exclusive enteral nutrition.

#### *Titration to target therapy*

EEN can be commenced at target volume on day 1 of administration if a polymeric formula is used. Gradual upgrade of formula over a 2–7‐day period may be required if an elemental formula is used as these are less palatable and can be poorly tolerated. Titration of therapy may also be required in those with problematic vomiting or diarrhea or are at risk of refeeding syndrome.^1^, ^18^, ^38^, ^39^, ^40^

### *Statement 4: Monitoring adherence and response while on EEN therapy*

1. All members of the IBD multidisciplinary team (MDT) should encourage and support adherence to EEN therapy (level 7 evidence).
2. Regular monitoring of EEN should be undertaken by the IBD team (level 7 evidence).
3. If EEN therapy does not induce clinical response within 2 weeks, an alternative treatment should be considered (level 7 evidence).

#### *Promoting adherence*

Nonadherence to EEN is a well‐documented barrier to achieving remission induction with EEN in adults.^2^, ^3^ Potential strategies to promote treatment adherence are outlined in Table 1.^2^, ^3^, ^4^

#### *Response to therapy*

Like any medical intervention, EEN therapy needs to be monitored and adjusted to ensure optimal efficacy (Table 3). Given that symptoms and actual inflammatory burden correlate poorly in patients with IBD, objective monitoring of disease activity is imperative to interpreting therapeutic efficacy.^41^ Although the endoscopic evaluation of luminal disease activity is considered the gold standard, biomarkers of inflammation are a useful surrogate means of measuring objective disease activity. FC is a sensitive measure of luminal inflammation responsive to therapy.^41^ Patient‐reported outcomes and IBD quality‐of‐life tools may also be beneficial to monitor outcomes of EEN therapy.^42^

**Table 3:** Monitoring criteria for EEN therapy

Nutrition prescription	Anthropometric measurements, biochemical nutrient studies, and tolerability of formula should inform recalculation of nutritional requirements and prescription
Inflammatory markers	Intestinal inflammation can be monitored using biomarkers of inflammation, C‐reactive protein, and fecal calprotectin
Clinical progress	Changes in disease activity can be semiquantified using tools such as CDAI or HBI to monitor effectiveness of therapy
Sonographic/radiological imaging	Imaging such as intestinal ultrasound or magnetic resonance enterography can be used to identify changes in disease activity pre‐ and posttherapy
Compliance	Adherence to complete EEN regimens should be reviewed and regimens individualized to promote compliance to duration of therapy.

CDAI, Crohn's disease activity index; EEN, exclusive enteral nutrition; HBI, Harvey Bradshaw Index.

#### *Early cessation*

In the absence of adult data, pediatric consensus guidelines recommend that EEN therapy should be ceased if no clinical improvement is seen within 2 weeks.^1^, ^13^ Where EEN is used for remission induction, commencing long‐term medical maintenance therapy should be a part of the initial shared decision‐making and establishment of treatment goals.^1^, ^13^

### *Statement 5: How to reintroduce food and wean EEN*

1. A normal diet can be reintroduced rapidly, one meal per day, following a prescribed course of EEN therapy (level 4 evidence).
2. An unrestricted, nutritionally balanced diet, tailored to patient preference, is recommended (level 7 evidence).
3. Reintroduction of EEN should be considered if there is a flare of disease during the food reintroduction phase (level 4 evidence).

#### *Reducing EEN*

**Figure 2:**
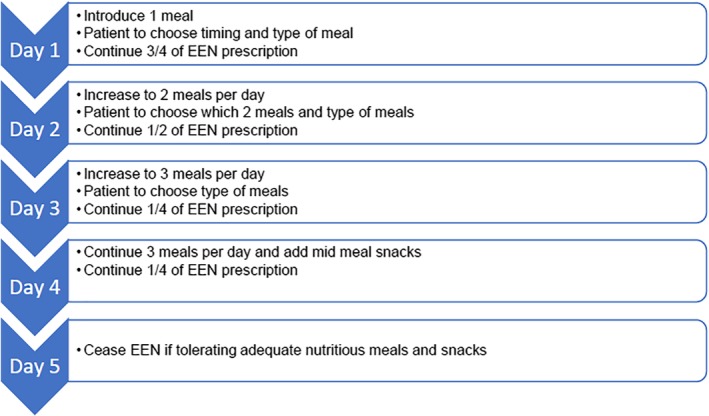
Weaning exclusive enteral nutrition formula and reintroducing food.

Formula can be reduced in volume and frequency, whilst food is reintroduced one meal at a time over a period of 5 days (Fig. 2).^43^

#### *Reintroducing food*

Food can be reintroduced at a rapid pace as tolerated by the individual without risk of reducing EEN efficacy (Fig. 2).^43^ If the individual is reluctant to reintroduce food rapidly, a slower approach can be taken to support emotional needs and balance nutritional intake.

#### *Diet after EEN*

A normal, unrestricted diet in line with national healthy eating guidelines should be the final goal of therapy when food is reintroduced.^1^, ^9^, ^44^, ^45^ Limited data propose three dietary options for food reintroduction: an unrestricted diet, an exclusion/elimination diet, and a low‐fat/fiber exclusion diet; however, it is unclear whether particular dietary components actually influence the success of food reintroduction after EEN therapy.^1^, ^34^

Dietary intake after EEN should be personalized by the dietitian and gastroenterologist, with consideration of stricturing disease, patient preference, comorbidities, motivation, and quality of life. If functional gastrointestinal symptoms persist when CD is in remission, an experienced IBD dietitian can provide additional dietary guidance.

#### *Recommencing EEN*

Retrospective pediatric data suggest that reinitiation of EEN should be considered if IBD disease activity flares during food reintroduction (57–77% efficacy); however, subsequent courses of EEN have lower treatment adherence.^46^, ^47^

### *Statement 6: The role of partial enteral nutrition in adults with active CD*

1. Partial enteral nutrition (PEN) cannot currently be recommended for the induction or maintenance of remission in adults with CD (level 7 evidence)

#### *PEN therapy*

Four published adult studies have used PEN to induce or maintain remission. The results are conflicting and limited by high dropout rates, different PEN regimens, and lack of control groups.^18^, ^34^ In pediatric and young adult CD cohorts, small studies have reported PEN with an exclusion diet to be effective in remission induction (70%) and maintenance of remission compared to an unrestricted diet (36.4 *vs* 64% relapse rate, respectively).^10^, ^48^, ^49^, ^50^ Pediatric guidelines do not recommend PEN for induction of remission but highlight its potential role as a maintenance therapy in mild disease.^10^, ^13^ Further research is required before a recommendation for PEN in adults with CD can be made.^34^

## Conclusion

The evidence for using EEN as a treatment option for inducing remission in adults with active CD is increasing and is supported by pediatric data. In adults who are adherent to EEN, there is no mechanistic reason, including disease duration or disease distribution, to conceive that efficacy would differ from pediatric cohorts.^2^

Corticosteroids and biologic therapies are currently more commonly used as first‐line therapy in adult populations^1^; however, the development of an optimal care pathway and patient resource provides clinicians with a protocol to offer patients EEN as an alternative therapy with holistic benefits. The development of this optimal care pathway for using EEN in adults with CD is an important step forward in providing a standard against which to benchmark care, so more robust data can be collected on the efficacy of using EEN as a therapeutic option.

EEN prescriptions should be individualized based on clinical indication identified by a multidisciplinary IBD team and a comprehensive nutritional assessment by a specialist IBD dietitian. Food should be reintroduced liberally with the long‐term goal of an individualized, unrestricted diet. Future multisite randomized controlled studies are required to evaluate the efficacy of using EEN as a therapeutic option in adults with CD.
